# Innovations in non-communicable diseases management in ASEAN: a case series

**DOI:** 10.3402/gha.v7.25110

**Published:** 2014-09-22

**Authors:** Jeremy Lim, Melissa M. H. Chan, Fatimah Z. Alsagoff, Duc Ha

**Affiliations:** 1 Health and Life Sciences Practice, Asia Pacific Region, Oliver Wyman, Singapore; 2 Vriens and Partners, Singapore; 3 Secretarial and Coordination Division, Cabinet Office, Ministry of Health, Hanoi, Vietnam

**Keywords:** non-communicable diseases, innovations, health care, developing countries, ASEAN

## Abstract

**Background:**

Non-communicable diseases (NCDs) are reaching epidemic proportions worldwide and present an unprecedented challenge to economic and social development globally. In Southeast Asia, the challenges are exacerbated by vastly differing levels of health systems development and funding availability. In addressing the burden of NCDs, ASEAN nations need to fundamentally re-examine how health care services are structured and delivered and discover new models as undiscerning application of models from other geographies with different cultures and resources will be problematic.

**Objective:**

We sought to examine cases of innovation and identify critical success factors in NCD management in ASEAN.

**Design:**

A qualitative design, focusing on in-depth interviews and site visits to explore the meanings and perceptions of participants regarding innovations in NCD against the backdrop of the overall context of delivering health care within the country's context was adopted.

**Results:**

In total 12 case studies in six ASEAN countries were analysed. Primary interventions accounted for five of the total cases, whereas secondary interventions comprised four, and tertiary interventions three. Five core themes contributing to successful innovation for NCD management were identified. They include: 1) encourage better outcomes through leadership and support, 2) strengthen inter-disciplinary partnership, 3) community ownership is key, 4) recognise the needs of the people and what appeals to them, and 5) raise awareness through capacity building and increasing health literacy.

**Conclusions:**

Innovation is vital in enabling ASEAN nations to successfully address the growing crisis of NCDs. More of the same or wholesale transfers of developed world models will be ineffective and lead to financially unsustainable programmes or programmes lacking appropriate human capital. The case studies have demonstrated the transformative impact of innovation and identified key factors in successful implementation. Beyond pilot success, the bigger challenge is scaling up. Medical technologies are crucial but insufficient; passionate and engaged leaders and communities enabled by enlightened policy makers and funding agencies matter more.

Non-communicable diseases (NCDs) have been described as presenting an unprecedented challenge to economic and social development globally (1, 2). The latest projections from the World Health Organization (WHO) suggest 57 million deaths occurred globally in 2008, of which 36 million (63%) were the result of NCDs. Despite the immense burden of disease, NCDs, defined here as cardiovascular diseases, cancers, chronic respiratory conditions, and diabetes (3), nonetheless present a public health opportunity to intervene, not just in disease treatment but at all stages of disease progression. It is estimated that 80% of NCDs are preventable with appropriate diet and lifestyle choices (4) and large-scale clinical trials such as the UK PDS (5) highlight that good control of NCDs can have substantial effect on the incidence of downstream complications.

ASEAN which comprises mainly emerging economies faces difficulties in combating NCDs related to the rapid increase in disease incidence and inadequate health systems preparation for these increases. For example, in Indonesia, 7.6 million people are living with diabetes, while another 12.6 million have pre-diabetes (6). By 2030, the number of people with diabetes in Indonesia will hit 11.8 million (7). This equates to a 6% annual growth that exceeds the country's overall population growth (8). Moreover, fewer than half of those with diabetes are aware of their condition (6) and likely many are poorly informed of the risk factors and the appropriate behaviours to mitigate the risk. Those who remain in rural areas will have the greatest need for treatment from a health care system strained by demand for resources (9). Developing countries in ASEAN also have to deal with the continuing challenges arising from communicable diseases. Unlike in the developed world, where infectious diseases such as malaria have largely been eradicated or controlled, developing countries face an unfortunate double burden of managing infectious diseases and the NCD epidemic, which is due in part to the rapidly changing lifestyles and diets over one generation (10). Hence, a comprehensive and coherent NCD programme has to be implemented at the same time as infectious diseases are being fought (11).

## The need for NCD health care innovation in ASEAN

According to the WHO, without intervention, deaths from NCD are predicted to increase by 15% between 2010 and 2020 (1). The biggest increase would occur in the African, Eastern Mediterranean, and Southeast Asian regions (1). Fortunately, most premature deaths from heart disease, stroke, and diabetes can be averted with behavioural modifications and pharmaceutical interventions (12).

Chronic diseases tend to be of long duration, can be progressively disabling, and are often life-threatening, requiring a much different model of health care than what most ASEAN countries have now (13). Our health care systems have been geared towards treating acute illnesses, which usually have an identifiable single cause and readily apparent cure. On the other hand, chronic diseases by their very nature require holistic interventions in lifestyle and behaviour, education in self-management, and complex life-long treatments. Hence, more of the same interventions based on episodic care models will not do (14).

ASEAN needs to fundamentally re-examine how health care services are structured and delivered while also being aware of the resource availability of well-resourced compared to low-resourced countries. Constrained in various ways including the shortage of skilled staff in remote settings, poor facilities, and scarcity of essential equipment and medicines, health care providers in low-resourced countries are compelled to innovate and seek practical solutions that may never have been thought of in the well-resourced developed world.

It is in this light that the ASEAN NCD Network sought to find examples of innovation and success factors of these innovations in NCD management in ASEAN. This was undertaken as part of a White Paper by the ASEAN NCD Network exploring the use of innovations in ASEAN. The ASEAN NCD Network is an informal grouping of health care experts from around the region working together to address the issue of NCDs. The network aims to promote regional collaboration and cross-country sharing of ideas and best practices within the ASEAN region.

## Objectives of the study

The paper does not set out to be a comprehensive plan for addressing the challenges of NCDs. It concentrates on innovation and what has worked in the ASEAN context. It highlights the awareness of the cultural context and sensitivity that is needed in introducing innovations in Asia. While the word ‘innovation’ often brings to mind highly complex and extremely costly technological advancements, ‘innovation’ in this context is not defined as highly complex technological advancements but encompasses also solutions that make use of cheap and readily available resources that are contextually relevant in the respective setting.

## Methods

### Design

We used a qualitative design, focusing on in-depth interviews to explore the meanings and perceptions of participants regarding innovations in NCD against the backdrop of the overall context of delivering health care within the country's context.

### Participants and setting

A purposive sampling was used to identify the cases for innovation with two distinct strategies used to generate a diverse pool of innovation cases. First, we circulated an innovation identification checklist (Table 1) to members of the ASEAN NCD Network Steering Committee (Table 2), personnel from the various ASEAN Ministries or Departments of Health, and global NGOs for recommendations. This was then followed up by a call to the respective informants to gather more details of the project leader information and to also verify that the participants met the checklist criteria.

**Table 1:** Checklist for innovation identification

Categories		Tick where applicable	Comments (if any)
1	Target one of four NCD focus areas	[ ]	_______
	Cardiovascular diseases	[ ]	_______
	Diabetes	[ ]	_______
	Chronic lung diseases	[ ]	_______
	Cancer	[ ]	_______
2	Types of prevention	[ ]	_______
	Primary	[ ]	_______
	Secondary	[ ]	_______
	Tertiary	[ ]	_______
3	Occurs within Southeast Asia	[ ]	_______
	Yes	[ ]	_______
	No	[ ]	_______
4	Programme duration ≥18 months	[ ]	_______
	Yes	[ ]	_______
	No	[ ]	_______
5	Reasonably high-quality data available	[ ]	_______
	Yes	[ ]	_______
	No	[ ]	_______

**Table 2:** Steering committee members

Dr Jeremy Lim, Partner, Head of Health and Life Sciences Practice, Asia Pacific, Oliver Wyman
Dr Tai E Shyong, Head, Division of Endocrinology, National University Hospital, Singapore
Dr Daphne Khoo, Chief Medical Officer, Fortis Healthcare International, Singapore
Dr Nazeli Hamzah, President, Malaysian Association of Adolescent Health, Malaysia
Dr Wasista Budiwaluyo, Secretary General, Indonesian Hospital Association (PERSI), Indonesia
Dr Ha Anh Duc, Senior Researcher, Division of Non-Communicable Diseases, Institute of Population, Health and Development (PHAD), Vietnam
Dr Ly Ngoc Ha, Director, Development Center for Public Health, Vietnam
The late Dr Alberto G. Romualdez Jr., President, Culion Foundation, Philippines
Dr Antonio Dans, Professor, College of Medicine, University of the Philippines Manila, Philippines
Dr Pura Angela Wee, Associate Director, Zuellig Center for Asian Business Transformation, Asian Institute of Management (AIM), Philippines

The process yielded 12 cases in six ASEAN countries, comprising Thailand, Singapore, the Philippines, Indonesia, Vietnam, and Malaysia.

### Data collection

The interviews were conducted from January 2013 through December 2013 by a multidisciplinary team with varied backgrounds and experience, including: clinical medicine, clinical epidemiology, and public health.

For each of the innovations identified for more study, the research team conducted preliminary phone or email interviews with the project leaders to ascertain the suitability of the case study based on the criteria checklist.

A question guide was developed for the study trips where the researchers would personally meet and interview them about their innovations. The topic guide covered open-ended questions seeking to understand their innovations, personal motivations, and challenges faced.

The research team also travelled to the innovation sites to observe the innovations in action and to speak to health care providers, volunteers, and patients on the ground for their own perspectives on the programmes.

### Analysis and validation

A thematic analysis approach was adopted. The analysis sought to identify associations between themes and report patterns (15). All interviews were recorded through note taking. The data was examined manually and the themes were identified by highlighting the hard copies. The thematic analysis was further tested during discussions amongst the study team.

## Results

### Basic characteristics of the case studies of innovation

In total 12 case studies on innovation were found in six ASEAN countries, categorised into three interventions: primary, secondary, and tertiary. Primary interventions accounted for five of the total cases, whereas secondary interventions comprised of four, and tertiary interventions three (Tables 3–6).

**Table 3:** Classification of case studies yielded from different countries

	Level of prevention			
		
	Primary	Secondary	Tertiary	Total
Thailand	1	0	1	2
Singapore	1	1	0	2
The Philippines	2	0	0	2
Indonesia	0	1	2	3
Vietnam	0	1	0	1
Malaysia	1	1	0	2

**Table 4:** Summary of five innovations on NCD primary prevention in ASEAN^a^

**Innovation 1. Lifestyle Building Programme, Theptarin Hospital, Thailand**	
Focus	Diabetes and related chronic diseases
Innovation	First hospital in Thailand to introduce integrated diabetes care team comprising of an endocrinologist, a diabetes nurse educator, and a dietitian; introduction of new care models through a paradigm shift
	Chronic diseases through healthy living and behaviour modification
Strategy	Education, research, and patient education to introduce the concept of holistic team-based care for patients with diabetes
Key finding	Commitment to public education and emphasis on professional capacity building
**Innovation 2. Strategies to Promote Healthy Eating Behaviours, Singapore Health Promotion Board**	
Focus	Obesity
Innovation	Encouraging the people of Singapore to eat healthy by using novel tools adopted by market research companies, and engaging local food manufacturers and hawkers to develop and sell healthier foods though the board's ‘Ask For’ program
Aim	Find innovative ways to promote health and healthy eating by empowering Singaporeans to request and opt to choose healthier foods when eating out
Strategy	Advancing health promotion initiatives upstream by engaging local food manufacturers to co-develop healthier foods
Key finding	Upstreaming of education, health promotion initiatives and interventions as shown by successful health programs promotion at the national level
**Innovation 3. Enactment of a ‘Sin Tax’ on Tobacco Products, Department of Health, Philippines**	
Focus	Tobacco use
Innovation	Utilisation of strategic means and political will by pro-sin tax reformers to push forth their agenda; signed into law in December 2012 after 16 years of review and revisions
Aim	Introduction of the Sin Tax law in the Philippines so as to reduce tobacco consumption
Strategy	Building a critical mass base for supporting efforts of tobacco control advocates and pushing of the tobacco tax law through engaging lawmakers’ support and civil society participation
Key finding	The confluence of a popular administration with a firm political will and closely coordinated actions between the policy holders and the tobacco reform advocates from the civil society is crucial in mobilising supporters from the government and people to bring about change
**Innovation 4. ‘Gotong Royong’ Behavioural Change Model, Melaka State Health Department, Ministry of Health, Malaysia**	
Focus	Obesity and tobacco use
Innovation	Using the concept of ‘gotong royong’ – the spirit of volunteerism, selflessness, and working together for the benefit of the community; villagers of Malacca were given RM4,000 (approx.US$1,250) per village to print materials, train volunteers in health education, nutrition, and exercise
Aim	Engaging villagers in Malacca to take charge of their health
Strategy	Identifying village heads who are keen in leading the project and providing support and education to train the volunteers
Key finding	Sense of ownership, community empowerment, and a spirit of volunteerism were important factors in making the community health programme a success
**Innovation 5. Package of Essential Non-Communicable Disease (PEN) Implementation, Department of Health, Philippines**	
Focus	Tobacco use and diet
Innovation	Implementation of the WHO PEN programme with modifications on strategies and techniques to fit the needs of the people of Pateros, Philippines
Aim	Reduction of premature deaths due to NCDs in Pateros
Strategy	Soliciting support for the implementation of the PEN project by engaging key stakeholders in the health care system and policy makers in Pateros
Key finding	The anecdotal evidence of increased awareness regarding NCDs as well as the programs is encouraging; the Department of Health has adopted the PEN guidelines and is considering scaling the program to the whole country

a Complete case studies can be found at: www.healthspace.asia.

**Table 5:** Summary of four innovations on NCD secondary prevention in ASEAN^a^

**Innovation 1. Visual Inspection with Acetic Acid (VIA), Female Cancer Programme, Indonesia**	
Focus	Cervical cancer
Innovation	Working within the constraints of low-resource settings, health care providers are compelled to innovate and seek practical solutions for cervical cancer screening
Aim	Introduction of cervical screening to the provinces in Indonesia
Strategy	Introducing creative low-cost cervical cancer screening treatment to the provinces in Indonesia as well as the significance of socialisation and education of the women and husbands on the need for cervical screening and early treatment
Key finding	Apart from the solutions, a can-do attitude, inventiveness, and true community spirit are needed to make a programme successful
**Innovation 2. Earlier Presentation of Cancer Patients for Definitive Diagnosis and Treatment, Department of Radiotherapy, Oncology, & Palliative Care, Sarawak General Hospital, Malaysia**	
Focus	Breast and nasopharyngeal cancer
Innovation	Creative means of utilising the medical assistants (MAs) and nurses in the rural clinics to diagnose the patients are adopted for better outcomes
Aim	Education of community nurses and MAs in the rural areas on early symptoms of cancer (breast, cervical, and nasopharyngeal)
Strategy	Training the MAs and nurses facilitating referral system from the rural area to the hospital
Key finding	Localisation of health education materials helps the rural villagers to understand and connect
**Innovation 3. Community-Based Hypertension Management, Vietnam National Heart Institute**	
Focus	Hypertension
Innovation	Strategic utilisation of human capital to ensure the success of the programme
Aim	Introduction of community-based hypertension management programme in Vietnam
Strategy	Engaging in an community-based lifestyle study
Key finding	The success of the program is contributed by 1) successful engagement of the whole community, 2) support from committed local authorities and medical expertise, and 3) training of committed health care workers
**Innovation 4. Tele-Health Interventions to Chronic Patients, Eastern Health Alliance, Singapore**	
Focus	Diabetes
Innovation	Development of an alternative care delivery methods for chronic patients post discharge using tele-health system used by the Eastern Health Alliance for its Health Management Unit
Aim	Introduce innovative ways to keep the aging population healthy and prevent them from hospital admission
Strategy	Identifying discharged chronic patients through the Relationship Management Program; the system monitors the health of the patient and alerts the health care provider if the patient's test results showed worsening condition, visiting for consult or missing a medical appointment will be monitored as well
Key finding	Alternative methods are needed to explore care delivery for patients while also being aware of the patient's needs during their illness journey

a Complete case studies can be found at: www.healthspace.asia.

**Table 6:** Summary of three innovations on NCD tertiary prevention in ASEAN^a^

**Innovation 1. Digital Retinal Photography for Diabetics in Rural Communities, The Center of Excellence (COE) for Retina Diseases, Rajavithi Hospital & Institute of Medical Research and Technology Assessment (IMRTA), Department of Medical Services, Ministry of Public Health, Thailand**	
Focus	Diabetic retinopathy (DR)
Innovation	Alternative solutions are explored to bring DR screening into the villages so that more people could benefit
Aim	Bringing eye care to the villagers so that villagers could have DR screening access
Strategy	Identify committed village volunteers who are not medically trained to learn and perform the DR screening
Key finding	A strong political will is needed to implement the Diabetic Blindness Prevention project to the community
**Innovation 2. Improving Care of Pediatric Patients with Diabetes, Indonesian Pediatric Endocrinology Chapter, Indonesian Pediatric Society (IDAI) and Indonesian Association of Families with Diabetes Mellitus Children (IKADAR)**	
Focus	Diabetes
Innovation	The project lead skilfully capitalised on data to seek funds and also utilised multi-prong approaches for outreach programmes to create awareness
Aim	Establish a comprehensive diabetes management programme for children with type 1 diabetes
Strategy	Identifying the childhood diabetes problem with data, reach out to the funding bodies to present the case and seek funding; building capacity among health care providers and engaging stakeholders and family in awareness programmes
Key finding	Education and awareness creation for the patients and convincing stakeholders are key to successful programme implementation
**Innovation 3. Community Diabetes Strengthening, Indonesia**	
Focus	Diabetes
Innovation	Flexibility and stakeholder engagement strategies to introduce the programme were highlights of the initiative
Aim	Improve the capacity of preventing, detecting, and treating diabetes to reduce the burden of diabetes in Indonesia
Strategy	Establishing diabetes management systems in hospitals and primary health centres in the community
Key finding	Results showed a 15% increase in diabetes education provided in the provincial hospitals and over 20% increase in the puskesmas. A total of 1,237 health professions in all were trained in diabetes management; establishment of specific diabetes clinics

a Complete case studies can be found at: www.healthspace.asia.

### Analysis

Five themes which contribute to successful innovation for NCD management were identified based on the themes gathered from the case studies.

### Theme 1: Encourage better outcomes through leadership and support

The importance of leadership to the change management process is underscored by the fact that change, by definition, requires creating a new system and then institutionalising the new approaches. Change leadership is needed to be the driving force to instill visions and processes that fuel large-scale transformation (16).

The case studies illustrate that successful programs at least initially depend on the people and the leaders who are driving and implementing the programs. Similar leadership traits were identified in the people who drove the programmes.

These programme leaders were adept at:

1. Identifying both internal and external strengths and weaknesses of themselves and the programmes
2. Influencing and mobilising team members and partners to complement their skills
3. Utilising their strong networks to support their vision

Above all, passion, vision, and a strong sense of mission to improve the lives of people were the driving force behind innovation in NCD management in these case studies.

Table 7 illustrates examples of how better outcomes are encouraged through leadership and support.

**Table 7:** How better outcomes are encouraged through leadership and support

Barriers or challenges	Solution	Leadership traits exhibited
**Example: Innovation 1 – Thailand**		
The ‘Innovator’ lacked experience and had no concrete plan in disease detection programmes Limited health care staff: The Thai government focus then was on treatment and prevention of acute diseases, rather than on chronic diseases such as diabetes. Hence, medical manpower was trained accordingly	Partner like-minded clinicians and hospital managers Serve as President of the Nutrition Association and Organising Chairman of the International Congress of Nutrition (2009) to inspire the establishment of a master's and an undergraduate programme in Food and Dietetics at Thai universities	• The ‘innovator’ was able to identify strengths and weaknesses • Mobilise the right people to complement strengths and weaknesses • Utilise networks to advocate for change
**Example: Innovation 7 – Malaysia**		
Nurses and medical assistants (MAs) were unable to refer patients with cancer symptoms to the district hospital due to the referral system limitations Doctors in the district hospital do not see patients who were referred by the nurses and MAs. This affects the nurses and MAs morale to refer patients	Change the workflow process in partnership with the State Health Department to allow nurses and MAs to refer patients directly Nurses and MAs would alert the programme leader directly so that action could be taken	• Utilise networks to advocate for change • Leadership commitment and support

### Theme 2: Strengthen inter-disciplinary partnerships

Collaboration is based on the understanding that individual and community well-being is determined as much by social, environmental, and economic systems as by health care provision. Hence, the promotion and maintenance of health does not belong to one professional group or sector. Partnership constructs are widely advocated in order to implement strategies to influence the wider determinants of health and health inequalities, and thus secure population health improvement. Partnerships are seen as important tools for improving public health outcomes because shared intelligence of both ‘soft’ and ‘hard’ information improves the understanding of the needs and wants of the local communities. It also provides opportunities for shared learning (17). Yet, it is also recognised that partnership is neither easy nor a panacea for tackling health issues. Central to partnership working is an awareness of different working cultures and the roles of individuals and professionals can influence outcomes of collaboration.

For participants to believe that the partnership is beneficial, a clear defined vision of what needs to be achieved has to be established (18, 19). Hence, taking time to identify shared values through open discussions is deemed to be the first step in partnership. For example, to solicit support for the implementation of the Package of Essential NCD interventions in Pateros, the project leader engaged key stakeholders in the health care system and policy makers in Pateros. The consultation workshop involving the key stakeholders helped to establish a defined goal. The mayor of Pateros was also advised on the growing economic and social burden of NCDs in Pateros, paving the way for securing the necessary funding.

In the community diabetes innovation in Indonesia, the project leader also recognised that strengthening government partnership is also important hence one of the key strategies is to continually advocate at all levels of the government.

### Theme 3: Community ownership is key

Community ownership contributes to effective initiatives. NCD prevention and treatment generally requires patients to alter entrenched behaviours. Medical professionals, however, have limited bandwidth and resources to consistently monitor patients. As such, community members play an important role in serving as doctors’ conduits to support and promote lifestyles that mitigate NCDs.

In Malacca, whole communities are educated on caloric counting and ways to prepare healthier foods. The village chief is an example of a health ambassador who serves to inspire the entire village. The community in Kampung Pantai Peringgit comes together to exercise by walking while at the same time inspecting and removing potential breeding sites for the *Aedes* mosquito, which transmits dengue to humans.

The importance of community ownership was also recognised by Dr. Paisan Ruamviboonsuk, project leader in ‘Digital Retinal Photography for Diabetics in Rural Communities’ who brought eye care to people in the rural areas of Thailand. He articulated, ‘One of the most important elements that made this initiative a success was giving an opportunity to local community health care personnel in rural areas, who were not trained in ophthalmology at all, to solve a problem in public health ophthalmology for their own people. They run their own project and they can do it successfully’.

### Theme 4: Recognise the needs of the people and what appeals to them

To garner support and sustain results, programmes must incentivise and appeal to all stakeholders involved.

Examining the success of the Vietnamese blood pressure control program, the consistent monitoring and measurements of progress were important factors that helped to sustain the program. Dr. Quang Ngoc Nguyen, cardiologist at the Vietnam National Heart Institute and project leader, emphasised, ‘We had funding for only a few years to pay for the high blood pressure medicines. After that, the villagers had to pay for the medicines out of their own pockets, and they did! And this was because we measured. They could see for themselves the difference taking medicines made to their blood pressure and this encouraged them to continue’. The element that contributed to the program's success was measuring and associating health and well-being to the villagers’ adherence to medication.

In another example of a national initiative to modify eating behaviours and encourage Singaporeans to eat healthily, the Health Promotion Board (HPB) worked with hawkers to launch Singapore's ‘Healthy Hawker’ initiatzive (20). To garner participation from the hawkers, HPB structured and marketed the program so that hawkers could see tangible benefits. It was reported that hawkers had a tripling of sales of dishes made with brown rice and wholegrain noodles and more importantly, earnings went up by at least 10% thus encouraging hawkers to support the programme.

However, different communities are in varying stages of willingness and readiness to change. Hence, community engagement and needs identification plays an important role. To address this, Dr. Noraryana in Malacca presented village leaders with a menu of options to enable leaders to select and tailor programs to suit their own community's needs and interests.

### Theme 5: Raise awareness through capacity building and increasing health literacy

One recurrent theme throughout the case studies is the importance of education for capacity building among the health care professionals and patient education for patients.

In the example of introducing early cancer detection in Sarawak, the team was unable to reach out to the local villages to educate them on early cancer detection. However once they identified the maximum point of leverage – the medical assistants (MAs) and nurses who were highly regarded by the villagers, the outreach efforts were much more successful. The MAs and nurses lived in the villages hence they knew the villagers well and were able to establish rapport with them. By engaging and training the MAs and nurses who had a desire to upgrade their medical skills and knowledge, the programme's outreach expanded.

Increasing health literacy and awareness of disease conditions among patients was also a key strategy through most of the case studies. Patient education materials and methods were contextualised and customised to fit the rural villagers’ local norms. To raise awareness of diabetes among stakeholders and the general public, mass media campaigns were also conducted in Indonesia's diabetes program. Dr. Aman Pulungan, the project leader, had appeared on ‘live’ television speaking about childhood diabetes.

## Discussion

Four points are worth noting. First, there is no dearth of innovations in NCD management occurring throughout ASEAN countries, and there are myriad opportunities for ASEAN countries to learn from each other.

Second, a common observation from the cases is the frugality of innovation. For example, Vietnam's blood pressure control program works out to US$ 0.06 per villager; Malaysia's cancer control program costs US$ 9,250 a year. The Indonesian effort to improve diabetes health care delivery across eight sites in Java, Sumatra, and Sulawesi needed less than half a million US dollars from the World Diabetes Foundation (21). In ‘See and Treat’ programs to address cervical cancer, inexpensive household vinegar is the key ingredient.

However, more important than frugality is the third noteworthy issue which is the needed flexibility in deploying funds. We had identified earlier the importance of community engagement and local solutions, and this necessitates on-site adaptation to meet local needs. Hence, although funds can be secured nationally or at state-level, their use is local. One commonly expressed frustration was the restriction and conditions on the use of funding. For example, programmes received funds that allowed for commercially printing materials but not for purchasing computers, printers, and paper which would have been more cost-effective. We spoke to many leaders who highlighted examples of having to ‘work around’ funders’ conditions and obtaining funding from varied sources so that collectively, the programme's resource needs could be adequately covered.

The success of NCD management programmes depends on the empowerment of the local community and local leadership. The practical reality is that different communities have different ways of executing projects and hence adopt different ways of using funds. Funding agencies need to recognise this and strike that balance between appropriate governance and accountability and enabling recipients to work efficiently and expeditiously. Our case series suggests that the best funding agencies, whether governmental or non-governmental, are the ones that are actively involved in the program with an on-site presence and quick and easy approval processes for any variations from the submitted proposal.

It is critical that practitioners have the freedom to allocate these resources flexibly to avoid waste and inefficiencies. This is in some ways akin to start-up investing where investors bring funding and short- and long-term objectives but leave the management team otherwise largely alone to execute. The requirement to deploy resources as flexibly as possible is necessary given the rapidly changing market dynamic. In innovating in health programs, the process of executing often brings new learning. The opportunities to incorporate new learning into the program are important. Budget allocations and interim milestones should hence be flexible enough to allow for evolution and flexibility.

### Strengths of the study

This study presents innovative health promotion efforts towards NCD management. It highlights the need to be culturally aware when engaging stakeholders and the patient groups. More importantly, this study highlights a common trait identified throughout the whole case series – political will and strategic engagement of key stakeholders are the key factors for successful programmes implementation.

### Limitations of the study

Although we have cast the net widely in terms of participants, the sample may not represent the full spectrum of innovations in ASEAN. We acknowledge that there are challenges in inter-sectoral collaboration, and we do not attempt to simplify the complex nature of stakeholder engagement and collaboration. However, our main aim was to identify common themes that allow for successful innovations in the respective ASEAN countries.

## Conclusions

Innovation is vital in enabling ASEAN nations to successfully address the growing crisis of NCDs. More of the same or wholesale transfers of developed world models will lead to financially unsustainable programmes or programmes lacking appropriate human capital. The case studies have demonstrated the transformative impact of innovation and identified key factors in successful implementation. Beyond pilot success, the bigger challenge is growing from ‘seed to scale’. Medical technologies are crucial but insufficient; passionate and engaged leaders and communities enabled by enlightened policy makers and funding agencies matter.

In order to tackle the growing challenge of NCDs, we propose three recommendations to support innovations in NCD management:

### Build an Asian databank of innovations for addressing the growing burden of diseases arising from NCD

The innovation databank should become a first-call resource for ideas and solutions for ASEAN countries to counter NCD. Evidence on the effectiveness, value-for-money, and likely impact of the innovations could be made available online. Information should be free and publicly accessible. Innovations that are unable to show impact and sustainability will also be included as this could present as learning points and future references.

### Introduce platforms for innovators to engage with each other, funders, and policy makers

Providing platforms for networking would enable like-minded innovators to share resources and practices and to provide support for further innovations. The inclusion of funders and policy makers would also enhance mutual understanding and enable decision makers to understand the needs and challenges of the people on the ground when implementing programs.

### Support the NCD management innovators in bringing from SEED to SCALE and building up sustainable business models

The SEED to SCALE theory of social change promulgated by Future Generations (22) suggests that the most available and sustainable approach to scaling up successful pilots lies in redirecting how people apply their energies. The most valuable resource is not money but the energy and enthusiasm of the community which is created and reinforced by autonomy and ownership. Hence, supporting the innovators to determine their own priorities and focus on practical solutions would enhance momentum for change and solutions to fit local circumstances.
